# Short and Long Term Outcome of Bilateral Pallidal Stimulation in Chorea-Acanthocytosis

**DOI:** 10.1371/journal.pone.0079241

**Published:** 2013-11-05

**Authors:** Marie Miquel, Umberto Spampinato, Chrystelle Latxague, Iciar Aviles-Olmos, Benedikt Bader, Kelly Bertram, Kailash Bhatia, Pierre Burbaud, Lothar Burghaus, Jin Whan Cho, Emmanuel Cuny, Adrian Danek, Thomas Foltynie, Pedro J. Garcia Ruiz, Santiago Giménez-Roldán, Dominique Guehl, Jorge Guridi, Marwan Hariz, Paul Jarman, Zinovia Maria Kefalopoulou, Patricia Limousin, Nir Lipsman, Andres M. Lozano, Elena Moro, Dhita Ngy, Maria Cruz Rodriguez-Oroz, Huifang Shang, Hyeeun Shin, Ruth H. Walker, Fusako Yokochi, Ludvic Zrinzo, François Tison

**Affiliations:** 1 Service de Neurologie, CHU Bordeaux, Bordeaux, France; 2 Service de Neurologie, CH François Mitterrand, Pau, France; 3 Univ.Bordeaux-INSERM U862, Neurocentre Magendie, Bordeaux, France; 4 Unit of Functional Neurosurgery, Sobell Department of Motor Neuroscience and Movement Disorders, UCL Institute of Neurology, Queen Square, London, United Kingdom; 5 Neurologische Klinik und Poliklinik, Ludwig-Maximilians-Universität München, Munich, Germany; 6 Neurosciences, Alfred Hospital, Commercial Road, Melbourne, Victoria, Australia; 7 Van Cleef Roet Centre for Nervous Diseases, Monash University, Melbourne, Victoria, Australia; 8 Sobell Department of Motor Neuroscience and Movement Disorders, University College London, Institute of Neurology, Queen Square, London, United Kingdom; 9 Service de Neurophysiologie Clinique, CHU Bordeaux, Bordeaux, France; 10 Department of Neurology, University Hospital Cologne, Cologne, Germany; 11 Department of Neurology, Samsung Medical Centre, Sungkyunkwan University, School of Medicine, Seoul, Korea; 12 Service de Neurochirurgie, CHU Bordeaux, Bordeaux, France; 13 Department of Neurology, Fundacion Jimenez Diaz, Madrid, Spain; 14 Servicio de Neurología, Hospital General Universitario Gregorio Marañón, Madrid, España; 15 Neurology and Neurosurgical Department, Clinica Universidad de Navarra, Pamplona, Spain; 16 National Hospital of Neurology and Neurosurgery, Queen Square, London, United Kingdom; 17 Division of Neurosurgery, Toronto Western Hospital, University of Toronto, University Health Network, Toronto, Ontario, Canada; 18 Morton and Gloria Shulman Movement Disorders Centre, Toronto Western Hospital, University of Toronto, University Health Network, Toronto, Ontario, Canada; 19 Movement Disorders Center, Department of Psychiatry and Neurology, University Hospital Centre of Grenoble, Grenoble, France; 20 Department of Neurology, Mount Sinai School of Medicine, New York, New York, United States of America; 21 Avicenna Medical Center, New York, New York, United States of America; 22 Department of Neurology, University Hospital Donostia, Neuroscience Unit, BioDonostia Research Institute, San Sebastian, Spain; 23 Department of Neurology, West China Hospital, Sichuan University, Chengdu, Sichuan, China; 24 Department of Neurology, James J. Peters Veterans Affairs Medical Center, Bronx, New York, United States of America; 25 Department of Neurology, Tokyo Metropolitan Neurological Hospital, Fuchu, Tokyo, Japan; 26 Université Bordeaux, Institut des Maladies Neurodégénératives, UMR 5293, Bordeaux, France; 27 CNRS, Institut des Maladies Neurodégénératives, UMR 5293, Bordeaux, France; University of Iowa Carver College of Medicine, United States of America

## Abstract

**Background:**

Chorea-acanthocytosis (ChAc) is a neuroacanthocytosis syndrome presenting with severe movement disorders poorly responsive to drug therapy. Case reports suggest that bilateral deep brain stimulation (DBS) of the ventro-postero-lateral internal globus pallidus (GPi) may benefit these patients. To explore this issue, the present multicentre (n=12) retrospective study collected the short and long term outcome of 15 patients who underwent DBS.

**Methods:**

Data were collected in a standardized way 2-6 months preoperatively, 1-5 months (early) and 6 months or more (late) after surgery at the last follow-up visit (mean follow-up: 29.5 months).

**Results:**

Motor severity, assessed by the Unified Huntington’s Disease Rating Scale-Motor Score, UHDRS-MS), was significantly reduced at both early and late post-surgery time points (mean improvement 54.3% and 44.1%, respectively). Functional capacity (UHDRS-Functional Capacity Score) was also significantly improved at both post-surgery time points (mean 75.5% and 73.3%, respectively), whereas incapacity (UHDRS-Independence Score) improvement reached significance at early post-surgery only (mean 37.3%). Long term significant improvement of motor symptom severity (≥20 % from baseline) was observed in 61.5 % of the patients. Chorea and dystonia improved, whereas effects on dysarthria and swallowing were variable. Parkinsonism did not improve. Linear regression analysis showed that preoperative motor severity predicted motor improvement at both post-surgery time points. The most serious adverse event was device infection and cerebral abscess, and one patient died suddenly of unclear cause, 4 years after surgery.

**Conclusion:**

This study shows that bilateral DBS of the GPi effectively reduces the severity of drug-resistant hyperkinetic movement disorders such as present in ChAc.

## Introduction

Chorea-acanthocytosis (ChAc) is an autosomal recessive disease due to mutations of the *VPS13A* gene encoding for chorein [1]. It belongs to the neuroacanthocytosis syndromes, a heterogeneous group of genetically defined conditions, exhibiting neurological and neuropsychiatric disorders with red blood cell acanthocytosis [1,2]. ChAc is very rare, and typically presents as an adult-onset progressive disorder resembling Huntington's disease (HD) [1,2]. ChAc has however a slower progression, and displays some more or less specific clinical features such as tongue- and lip-biting, self-mutilations, seizures and neuromuscular manifestations but no specific neuropathological features besides prominent striatal degeneration [2,3].

There is no specific treatment for ChAc and the most frequent and disabling motor disorders, chorea and dystonia, are poorly controlled by conventional symptomatic drug therapy [2]. This has prompted neurosurgical treatment attempts, such as stereotactic brain lesions [4,5], and, more recently, deep brain stimulation (DBS) surgery of the internal globus pallidus (GPi) [6-8] following reported success in the management of other hyperkinetic movement disorders [9-12]. However, only single case or small series have been reported heterogeneously and in the short term, which likely bias the results towards favourable outcomes.

We performed a worldwide multicentre retrospective and cross-sectional study to gather all currently available data on all ChAc patients treated by GPi DBS and to provide a standardized, long-term comprehensive review of the clinical and functional outcome of these patients. In addition, to help guide GPi DBS surgery decision making in ChAc patients we aimed at identifying factors corresponding with favourable outcomes.

## Clinical Materials and Methods

A total of 12 centres were contacted to participate in this retrospective study. 

### Case selection

Movement disorders and neurosurgical tertiary centres were identified and contacted on the basis of scientific publications (original research and review articles published between 1997 and November 2012) in Medline and PubMed databases (search terms: deep brain stimulation, DBS, high-frequency stimulation, pallidal stimulation, and neuroacanthocytosis, chorea, chorea-acanthocytosis, ChAc), as well as of meeting abstracts. The support association for neuroacanthocytosis patients (www.naadvocacy.org), and the diagnostic reference centre and database in Munich, Germany, also collaborated for patient identification, and announcements were published at the Movement Disorders Society (MDS) meeting 2011 and MDS website. 

### Patients

Selected patients were diagnosed with molecularly proven or clinically probable ChAc (Table 1), and treated with GPi DBS regardless of surgery outcome. In the case of previously published cases, authors were asked to provide additional updated long term information at last patient visit using a standardized data collection sheet. Recruitment was conducted from February 2011 to November 2012.

**Table 1:** Basic diagnostic data in 15 patients with chorea.

**Patient**	**Acanthocytes (%)**	CK*	**Chorein in Western blot of red cells membranes**	***VPS13A* mutations**	**MRI (atrophy)**
**1**	20	x4	absent	ND	Ca, Pu, Cx
**2**	40	x4	absent	ND	Ca
**3**	10	ND	present (2 blots in 4 years)	present, o.m.	Ca
**4**	↑	x2.6	absent	present, c.h.	nl
**5**	25	x1.1	absent	present, H	Ca, Pu, Cx; striatal T2 hyper-intensity
**6**	31	x2.1	absent	present, H	Ca, Cx
**7**	ND	ND	ND	present, c.h.	nl
**8**	20	x1.6	ND	present, c.h.	Ca heads, Pu with T2 hyper-intensity
**9**	10	x18.8	ND	present, H	nl
**10**	↑	ND	subnormal low expression	ND	nl
**11**	20	x4.2	ND	present, c.h.	Ca heads, subcortical.
**12**	↑	ND	absent	ND	Ca heads, Pu T2 hyper-intensity
**13**	7	x13.2	absent	ND	Ca, lentiform nuclei with T2 hyper-intensity
**14**	6	x2.2	ND	ND	Ca, Pu, T2 hyper-intensity
**15**	6	ND	ND	ND	Ca, Pu, T2 hyper-intensity

CK = creatine kinase; ↑ elevated; * relative increase with respect to normal value for each centre laboratory; o.m. = one mutation known; c.h. = compound heterozygous; H = homozygous; Ca = Caudate; Cx = Cortical; Pu = Putamen; nl = normal; ND = not done.

### Data acquisition, protocol and surgery outcome measures

All centres were sent a standardized data collection sheet, prepared according to the guidelines for reporting results from clinical DBS studies in Parkinson’s disease [13].

Data were collected 2-6 months preoperatively (PREOP), at 1-5 months postoperatively (early post-operative: EPOP), and 6 months or more after surgery (last outcome reporting: LOR) (see Table S1).

Change in the severity of the motor disorder was measured by the Unified Huntington’s Disease Rating Scale-Motor Score (UHDRS-MS), and in functional status by the UHDRS-Independence Score (UHDRS-IS) and the UHDRS-Functional Capacity Score (UHDRS-FCS) [14]. In accordance with previous studies [9,15] an improvement equal or superior to 20% of the baseline score was considered as clinically relevant. 

### Data analysis

Data obtained are presented as the mean ± SEM values for each scale administered and at each time point: PREOP, EPOP and LOR. Statistical analysis was carried out using Statistica 8.0 for Windows (Statsoft, Maison-Alfort, France). Data were first analyzed by the Kolmogorov-Smirnov Test for normal distribution (p > 0.05). Thereafter, the effect of DBS upon each variable (UHDRS-MS, UHDRS-IS, UHDRS-FCS) was assessed by a multifactor analysis of variance (ANOVA) with time as the within-subjects factor. When ANOVA results were significant (p<0.05), the post hoc Bonferroni test was applied to allow adequate multiple comparisons between groups.

For each variable, ANOVA was performed using only those cases (11 patients) for which values were available at all three time points (Table 2). Individual values available for each patient were also entered and plotted at each time point. Data available at EPOP and/or LOR time points were also expressed as the percentage of PREOP values (100%).

**Table 2:** Number of patients with exploitable data.

	**PREOP**	**EPOP**	**LOR**
UHDRS-MS	13	13	11
UHDRS-FCS	14	14	11
UHDRS-IS	14	14	11
BFMDRS	1	1	1
MMSE	9	7	3
Adverse events	NA	15	15
Clinical status/outcome by symptoms	15	14	14
Drug treatment	14	-	14

PREOP: preoperative; EPOP: early post-operative; LOR: last outcome report; UHDRS-MS: Unified Huntington’s Disease Rating Scale-Motor Score; UHDRS-FCS: UHDRS-Functional-Capacity Score; UHDRS-IS: UHDRS-Independence Score; BFMDRS-M: Burke-Fahn-Marsden Dystonia Rating Scale-Motor part [16]; MMSE: Mini Mental State Examination [17]; NA: not applicable.

A linear regression analysis was performed to identify preoperative factors (age at surgery, disease duration and severity) that could predict DBS-induced improvement of motor and/or functional impairment at EPOP and/or LOR assessments. Analysis was performed using cases for which postoperative UHDRS values were available at either EPOP and/or LOR evaluations (see Table 2). “Improvement” was considered for each patient as the difference between UHDRS scores at PREOP and EPOP or LOR time points. P-values < 0.05 were considered significant; no adjustment was made for multiple testing.

### Ethics

The study was conducted after approval from the local Ethics Committee of the University Hospital of Bordeaux, and was carried out in accordance with the ethical standards laid down in the 1964 Declaration of Helsinki. All patients or a family member gave their written informed consent prior to their inclusion in the study, and data were entered into a database in an anonymous format in accordance with the personal data protection laws in France (CNIL).

## Results

Eleven out of the 12 centres (see Appendix S1) provided data. Fifteen patients were included in the data analysis but data for one patient (patient 11) were extracted only from a previous publication, and UHDRS scores were missing. Table 2 indicates the number of patients available for analysis at each time point. Twelve of the 15 cases have already been published in either scientific article format (n=9) or in congress posters (n=3) (for individual references see Table 3).

**Table 3:** Patient characteristics.

Patient number	Gender	Age at onset of symptoms (years)	Age at time of diagnosis (years)	Age at surgery (years)	Duration of disease (years)	LOR after surgery (months)
1 [18]	F	25	26	33	8	60
2	F	31	34	38	7	36
3	F	35	37	41	6	36
4 [19,20]	M	48	49	49	1	41
5 [21]	M	29	46	48	19	24
6 [21]	M	30	32	32	2	36
7 [22]	M	22	24	31	9	39
8 [23]	F	35	38	39	4	13
9 [24,25]	M	24	29	32	8	84
10	F	25	29	32	7	6
11 [26]	M	35	38	38	3	NA*
12 [8]	M	30	45	54	24	5
13 [8]	M	32	39	43	11	3
14 [7]	M	17	40	40	23	9
15 [7]	M	18	30	30	12	21
Mean ± SD	5 F (33%) 10 M (67%)	29.1 ± 7.8	35.7 ± 7.5	38.7 ± 7.3	9.6 ± 7.2	29.5 ± 23

F = female, M = male; LOR = last outcome report; NA = not available; * electrodes were removed 3 weeks after surgery.

### Patient clinical features

Patient characteristics are summarized in Table 3. Most of the patients presented with chorea (n=14, 93%) and/or dystonia (n=12, 80%), frequently associated with trunk spasms (n=12, 80%) and/or head drops/chorea (n=10, 67%), leading to recurrent face and head trauma. Most patients had gait and balance impairment. All patients had dysarthria, severe in 6. Orofacial dyskinesias and tics were present in 12 patients, 10 displayed lip-, tongue- or cheek-biting. Swallowing problems were present in 12 patients. Five patients had epilepsy, effectively treated in all. Psychiatric and behavioural disturbances, mainly depression and obsessive-compulsive behaviour, were present in 11 patients. Cognition was reported as slightly impaired in 6 patients, but extensive neuropsychological evaluations were lacking.

Surgery was indicated to reduce disabling motor symptoms such as chorea, dystonia, trunk spasms, falls, gait impairment (7 patients), self-mutilating and biting behaviours (6 patients) or head banging induced by trunk spasms or head drops (4 patients), feeding dystonia (3 patients) and recurrent belching (2 patients). 

### Outcome measures of global efficacy

**Figure 1:**
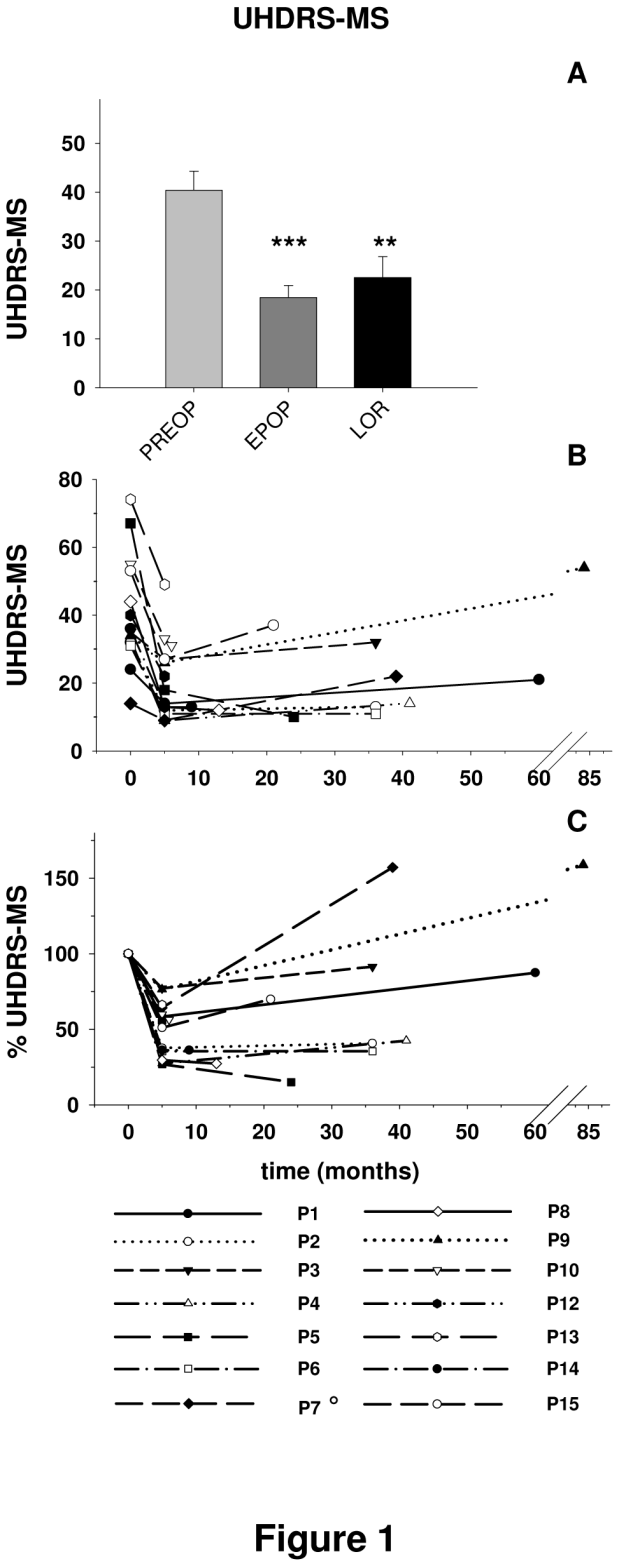
Effect of deep brain stimulation on motor impairment as assessed by the Unified Huntington’s Disease Rating Scale-Motor Score (UHDRS-MS). Bar histograms (panel **A**) represent means ± SEM (n= 11 patients). Line plots (panels **B**, **C**) show individual values per patient plotted at each of three time points: 2-6 months preoperatively (PREOP), 1-5 months postoperatively (early post-operative: EPOP), and 6 months or more after surgery (last outcome reporting: LOR). Individual data curves are shown in panel **B**, whereas panel **C** depicts percentage changes at EPOP and LOR with PREOP values set to 100% to make improvements and deteriorations easier to distinguish. Scores of patient 7 correspond to the Burke-Fahn-Marsden Dystonia Rating Scale-Motor part (BFMDRS-M) [16]. **p<0.01, ***p<0.001 *versus* PREOP time point (Bonferroni test after ANOVA: F_(2, 20)_= 15.11, p< 0.001, n= 11).

Figure 1 displays the effect of DBS on UHDRS motor scores at EPOP and LOR time points compared to baseline. Mean UHDRS-MS (Figure 1A) was significantly reduced at both time points after surgery. Mean proportional improvement of UHDRS-MS compared to baseline was +54.3% and +44.1% for EPOP and LOR, respectively. Figures 1B and 1C show individual outcomes on UHDRS-MS scores and changes compared to PREOP values. All 13 patients showed an improvement of 20% or more at EPOP, whereas at LOR, only 8 of these 13 patients (61.5 %), for whom UHDRS-MS was available, showed a significant improvement.

**Figure 2:**
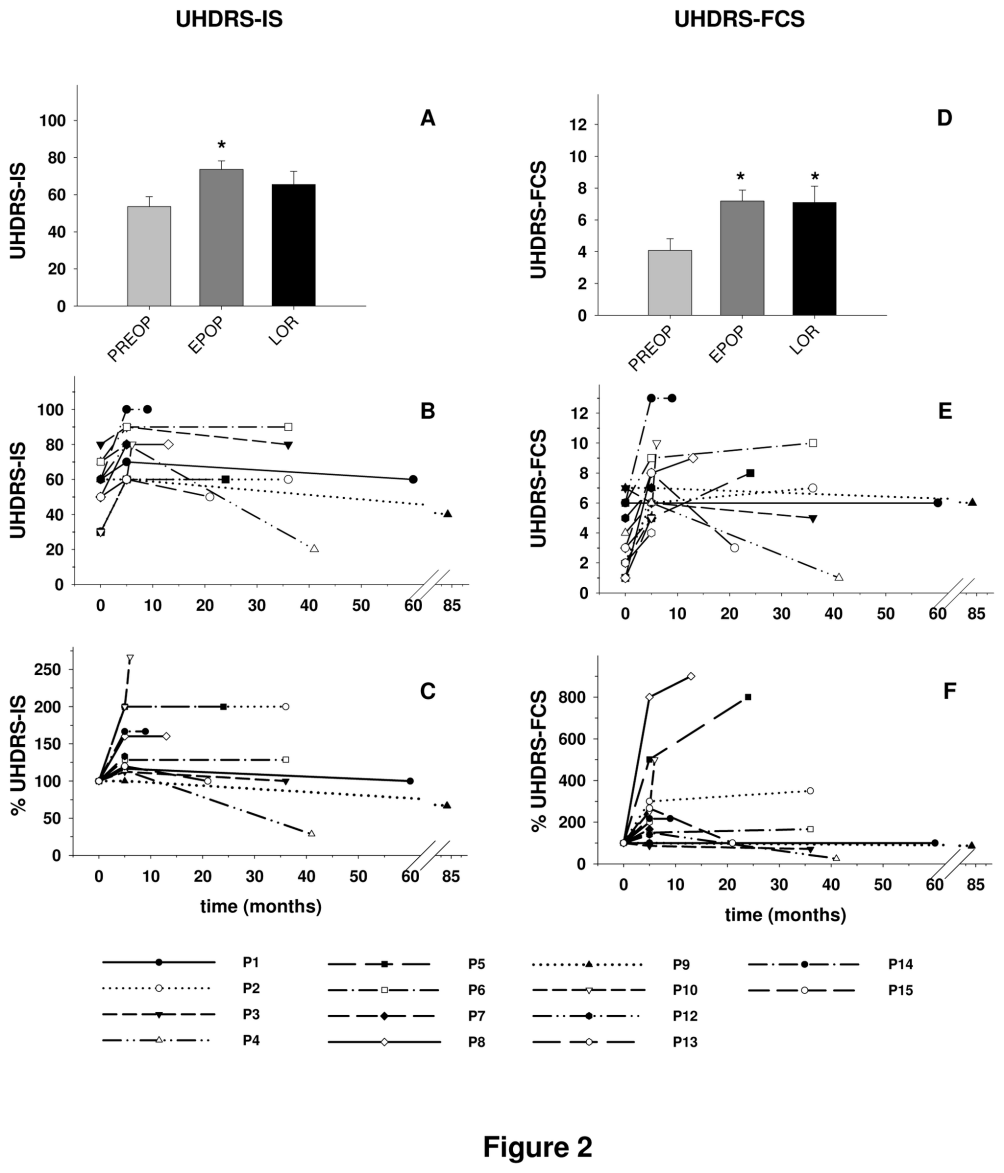
Effect of deep brain stimulation on independence and functional impairment as assessed by the Unified Huntington’s Disease Rating Scale-Independence Score (UHDRS-IS) and UHDRS-Functional Capacity Score (UHDRS-FCS), respectively. Bar histograms (panels **A**, **D**) represent means ± SEM (n= 11 patients). Line plots (panels **B**, **C**, **E**, **F**) show individual values per patient plotted at each of three time points: 2-6 months preoperatively (PREOP), 1-5 months postoperatively (early post-operative: EPOP), and 6 months or more after surgery (last outcome reporting: LOR). Individual data curves are shown in panels **B** and **E**, whereas panels **C** and **F** depict percentage changes at EPOP and LOR with PREOP values set to 100% to make improvements and deteriorations easier to distinguish. *p<0.05 *versus* the corresponding PREOP time point (Bonferroni test after ANOVA: F_(2, 20)_= 4.72, p< 0.05, n= 11, and F_(2, 20)_= 5.94, p< 0.01, n= 11, for UHDRS-IS and UHDRS-FCS, respectively).

DBS effects on the patients’ functional status, as evaluated by UHDRS-IS and UHDRS-FCS, are displayed in Figure 2. Mean UHDRS-IS was improved at both time points after surgery (EPOP: +37.3%, LOR: +22%), but reached significance only at EPOP (Figure 2A). Ten out of 14 patients (71.4 %) for whom UHDRS-IS was available at EPOP showed a significant improvement, which was still observed at LOR for 6 of them (Figure 2B, 2C). Mean UHDRS-FCS was significantly improved at both time points post-surgery (EPOP: +75.5%; LOR: +73.3%; Figure 2D). Eleven out of 14 patients (78.6 %) for whom UHDRS-IS was available at EPOP showed a clinically relevant improvement; which was still observed at LOR for 6 of them (Figure 2E, 2F).

### Outcome by symptoms

Chorea and dystonia were the most improved symptoms in a majority of patients. In terms of chorea, 13 out of 14 patients were improved at EPOP, observed within hours or days after surgery. At LOR, some relapse of choreic movements was observed in 3 patients (1, 4 and 15 at 24, 11 and 21 months, respectively), while the effect was maintained in others. Trunk spasms improvement was marked and maximal at EPOP for 11 out of 12 patients. Patients 3 and 9 experienced slower improvement of limb dystonia, with the maximum effect observed at LOR.

There was improvement of gait in patients with chorea and dystonia, but this was not significant in patients with pre-operative parkinsonism (4 out of 6). Except for patient 15, all patients who suffered from orofacial movements and self-mutilation improved. DBS had little effect on dysarthria, only 2 improved at EPOP, one of whom was found worse at LOR; one patient (patient 9) presented complete anarthria after surgery which improved after withdrawal of stimulation. Feeding status was available at LOR only in 9 patients and 6 had improved. Insufficient information was available to allow conclusions upon cognitive or neuropsychiatric and behavioural disorders. Mini Mental State Examination [17] was available in 9 patients before surgery (mean score = 26.8; range = 19-30), in 7 patients at EPOP (mean score = 27.3; range = 25-30), and in 3 patients at LOR (mean score = 26.7; range = 26-27). After surgery, antidepressant treatment remained unchanged in 6 patients. Mood improved for 3 patients, but no change was reported for the others. Four patients (patients 2, 7, 10 and 11) had preoperative compulsive behaviours: after surgery a marked and sustained (48 months) improvement was observed in one patient (patient 7).

### Relationship between preoperative status and surgery outcome

**Figure 3:**
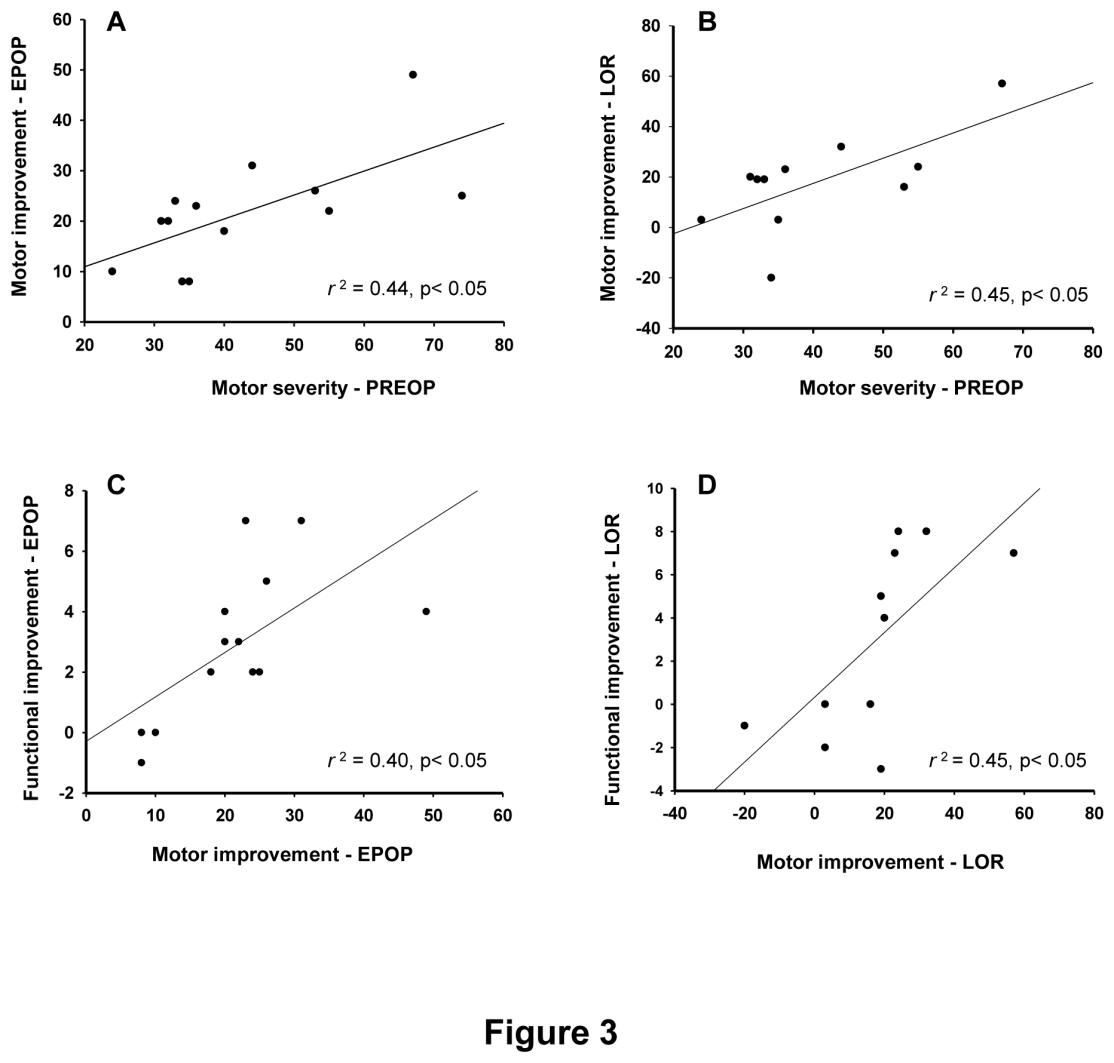
Regression analysis between preoperative (PREOP) severity of motor impairment, as assessed by the Unified Huntington’s Disease Rating Scale-Motor Score (UHDRS-MS), and motor severity improvement at early and late post-operative time points (EPOP, panel A, *r* = 0.66, *r* ^2^ = 0.44, p< 0.05, n=13; LOR, panel B, *r* = 0.67, *r* ^2^ = 0.45, p< 0.05, n=11); and motor severity improvement *versus* functional improvement, as assessed by the UHDRS-Functional Capacity Score (UHDRS-FCS), at EPOP (panel C, *r* = 0.63, *r* ^2^ = 0.40, p< 0.05, n=13) and LOR (panel D, *r* = 0.67, *r* ^2^ = 0.45, p< 0.05, n=11) time points. For each patient, motor and functional improvements correspond to the difference between appropriate UHDRS scores at PREOP and EPOP or LOR time points.

No significant relationship was found between age at surgery or disease duration and postoperative improvement on either motor or functional scales. Preoperative motor severity in UHDRS-MS predicted improvement in motor function at both EPOP (Figure 3A) and LOR time points (Figure 3B). Motor improvement was associated with an improvement in functional status (UHDRS-FCS) at EPOP and at LOR (Figure 3C, D); but not in the dependency score at both time points (UHDRS-IS).

### Surgery: implantation, target coordinates and stimulation parameters

Surgical procedure details and individual electrode coordinates as per the Schaltenbrand and Wahren atlas [27] are reported in Tables S2 and S3, respectively. Mean and range of stimulation parameters at per-operative, EPOP and LOR assessments are displayed in Table 4; individual optimal stimulation settings are given in Supporting Information, Table S4. 

**Table 4:** Mean stimulation parameters.

**Parameter**	**intra-OP (n=14)**	**EPOP (n=14)**	**LOR (n=12)**
Pulse frequency (Hz)	102.5 (40-185)	114.3 (40-185)	128.8 (40-185)
Pulse width (μs)	90.0 (60-210)	96.3 (60-180)	104.0 (60-150)
Pulse amplitude (V)	2.8 (1.6-4.5)	2.9 (2-4.5)	3.4 (2-4.8)

Data= mean (range); intra-OP= intra-operative; EPOP= early post-operative; LOR= last outcome report.

The pulse frequency applied at EPOP was high in 10 patients (130-185 Hz), and low in 4 (40-60 Hz). Stimulus duration, frequency and amplitude tended to increase at LOR. No patient initially treated with high frequency was switched to low frequency for further improvement; only one patient initially treated with low frequency was switched to high frequency stimulation at LOR (patient 9). At EPOP, stimulation was single monopolar for 10 patients and double monopolar for 4 patients. Most patients were treated via ventral contacts. At LOR, the stimulation field tended to be larger and more dorsal, 4 patients were still stimulated by single monopolar contact, and 6 by double or triple monopolar contacts. 

### Movement disorders medication

Before surgery, all patients took at least one drug for their movement disorder (neuroleptics, tetrabenazine, benzodiazepines, amantadine, anticholinergic drugs (mean number taken: 3 ± 1, mean number tried: 4 ± 2). At LOR, medication was reduced in 7 patients (50%) and had remained unchanged in 3 patients (21%), but drug number had increased in 4 patients (28.6%). In only one patient (patient 9) medication was discontinued for 2 years after surgery.

### DBS adverse events

Data on adverse events were available for all 15 patients. 

#### Surgery-related adverse events

Patient 9 had a misplaced right electrode requiring a new surgery. Two months after surgery, patient 13 had a device infection leading to implanted pulse generator (IPG) removal, thereby causing severe rebound of abnormal movements requiring sedation in the intensive care unit. Ten days later, while the infection was apparently resolved, a new IPG was implanted but subsequently the patient developed a cerebral abscess at the sites of the left electrode tracks, which prompted the complete removal of the whole system. One year later, a new right GPi DBS has had a positive effect on left-sided symptoms. 

#### Stimulation-related adverse events

Patient 1 displayed a worsening of trunk spasms at 40-50Hz stimulation frequency and with the use of the ventral contact he experienced gait freezing that disappeared with stimulation at more dorsal contacts but at the cost of worsening hyperkinesias and trunk spasms. Patient 4, at EPOP period, complained of abnormal face and mouth pulling over 60 Hz stimulation. One month after surgery he displayed increased tongue movements at 50Hz stimulation using bilateral double monopolar settings (C+1-2-), thereby requiring an adjustment towards bilateral single monopolar (C+1-) stimulation. Patient 9, at EPOP time point, exhibited worsening of chorea and dysarthria, with palilalia and drooling at 130Hz, 40Hz stimulation was effective on choreic movements. At LOR assessment, again under 130Hz stimulation, abnormal movements were well-controlled but clinical examination revealed mild parkinsonism and complete anarthria. Rigidity and bradykinesia slightly decreased 2 hours after stopping the stimulation, and the patient was able to pronounce few simple words. Patient 11 experienced increased chorea and side effects (facial hemispasm, visual disturbances and vegetative changes) whatever the bipolar stimulation parameters used at high frequency. Low frequency stimulation led to deterioration of speech and gait [26]. Patient 14 had an exacerbation of both chorea and dystonia at high-frequency (130 Hz) stimulation. Patient 15 complained of blurred vision during the per-operative macro-stimulation on the lowest plots and using high amplitude. 

#### Device-related adverse events

Two years after surgery, patient 2 experienced a “Twiddler syndrome” with lead rupture that required the replacement of the extension wires, IPG and both electrodes [28]. Device infection in patient 13 was discussed above.

#### Patient-related adverse events

At LOR, patient 4 requested to have the stimulator switched “off” because of suspected stimulation-related increased involuntary tongue protrusion. This, however, resulted in a further increase of tongue thrusting and DBS subsequently was again turned “on”, with settings unchanged. This patient suddenly died at home at age 53, 4 years after surgery and 1 year after LOR. An autopsy was not performed and the cause of death remained undetermined.

## Discussion

The present long-term multicentre study of 15 cases of ChAc treated with bilateral DBS of the GPi provides evidence that stimulation of the postero-ventrolateral "sensorimotor" portion of the GPi may help to manage severe, drug-resistant movement disorders, particularly chorea and dystonia. In the 11 patients, with two postoperative standardized evaluations, a significant long term improvement of motor symptom severity (improvement ≥ 20% in UHDRS-MS) was observed for 61.5 % at a mean follow-up of 2.5 years. Because duration of follow-up was variable, we constructed individual outcome curves that demonstrated that most patients remained above the pre-operative level, even though there was a tendency for motor deterioration over time. Gait impairment, orofacial movements and tics, self-mutilation, head drops and trunk spasms were DBS-sensitive symptoms, while dysarthria and feeding problems were less DBS-responsive and had a more variable outcome. Limb, face and trunk chorea improved early after surgery and responded immediately to switching the stimulation “on” and relapsed abruptly when switching “off”. This response resembles that observed in HD [10,29,30] and in McLeod Syndrome [5,25]. 

An early improvement was observed for phasic/mobile dystonia (debilitating trunk and limb spasms, head drops with injuries), as described in other conditions including primary generalized dystonia (PGD) [11,31,32]. Conversely, improvement of tonic dystonia was slower and occurred after 6 months of stimulation for patients 3 and 9, also resembling observations made in PGD [31,33].

As in other primary choreic syndromes [10,29] we observed a tendency for symptom deterioration and/or occurrence of parkinsonism in the longer term. This may be related to disease progression and/or to DBS itself, possibly by activation of inhibitory pallidal outputs to the motor thalamus [34]. Although GPi-DBS is an effective treatment for motor symptoms in Parkinson’s disease (PD), worsening of akinesia and gate deterioration was also observed in GPi-DBS-treated PD patients depending on the stimulated area within the GPi [34]. 

Dysarthria occurrence or worsening could also be related to disease progression or to DBS adverse effect, as observed in one of our patients (patient 9). GPi-DBS efficacy on swallowing was highly variable. Improvement could be seen when swallowing impairment was mainly due to feeding dystonia, orolingual movement disorders or trunk spasms. Otherwise, swallowing problems tended to deteriorate along with axial signs and parkinsonism. 

At LOR assessment, drugs used to control the movement disorders were reduced in 71% of patients (10/14), thus supporting the assumption that the effect upon movement disorders can reasonably be attributed to DBS rather than to drug regime changes. Only one patient did not benefit from strictly monopolar DBS. His disease was very advanced (17 years disease duration) as well as drug-resistant (11 drugs had been tried). 

Motor improvement translated into an improvement of functional capacity both at early and late post-operative observations. The patients showed significant functional improvement of 75.5% (UHDRS-FCS) at EPOP and LOR. Independence, as assessed by the UHDRS-IS, was significantly improved only at EPOP, even if there was also a trend for improvement at LOR. Thus, improvement in motor signs and functional capacity did not fully translate to improvement of dependency at the long-term. This could be due to the sensitivity and/or the psychometric characteristics of the subscale used and/or the low number of patients under study. Alternatively, DBS, while improving motor symptoms and function, may be ineffective with respect to dependency in patients severely affected by advanced disease. This issue remains to be addressed in prospective studies using also quality of life measures. The cognitive outcome of ChAc patients with GPi DBS was inconclusive with the limited data in hand. 

There was no correlation between the patients´ demographics or disease duration and outcome. However, there was a significant relation between pre-operative severity of motor signs (UHDRS-MS) and improvement of motor signs. 

The UHDRS scale was used as previously proposed for neurodegeneration with brain iron accumulation (NBIA) in an attempt to standardize the data available [15] but, as in NBIA, the UHDRS has not been validated in ChAc. Although the disease spectrum is very similar to HD, the scale may have missed characteristic ChAc symptoms such as orofacial movement disorders and self-mutilations, feeding dystonia, trunk spasms, head drops, breathing dystonia and recurrent belching. Formal validation of the UHDRS in ChAc or design of a ChAc specific scale is needed but difficult to perform due to the very small number of patients involved. 

Implanted devices and stimulation parameter settings varied among centres. However, optimal stimulation parameter settings mostly used higher frequency (mean = 102.5 Hz) and monopolar stimulation of lower contacts with a mean amplitude of 2.8 volts. There was a tendency to gradually increase frequency and amplitude along with the use of more dorsal contacts at the latest follow-up [35,36]. This may be due to disease progression or, alternatively, to an attempt to control increased bradykinesia over time [37].

The risk/benefit ratio appears acceptable in the present case series. Although the limited number of patients prevents definitive conclusions, adverse events were roughly similar to those observed in HD, PD, NBIA or dystonia case series [10,15,30,31,38,39]. Misplacement of one electrode requiring new surgery, Twiddler syndrome and device infection with brain abscess were the most serious surgery-related adverse events. In the 5 patients with preoperative epilepsy, there was no worsening of epilepsy or increase in antiepileptic drug dosage, and no *de novo* seizures after surgery in patients without epilepsy. These findings, although observed in few patients, suggest that epilepsy may not be a contraindication or a serious concern for GPi-DBS in ChAc, but further studies are needed to address this point. Indeed, a recent case reported possible intraoperative seizures and also post-operative intracranial hematoma in a 32-years-old patient with non-molecularly proven ChAc [40]. Two years later, the patient underwent a second DBS operation leading to a marked improvement of his dystonia, chorea and overall quality of life 2 and 8 months postoperatively [40].

Finally, although our results are positive and encouraging, it is important to note that our study presents some limitations due to its retrospective and multi-centre nature leading to some lack of homogeneity in patients’ assessment and data collection, despite of our efforts of standardization. Also, some data were missing (see Table 2), and in two patients (Table 1, patients 14 and 15) the diagnosis of ChAc was only “highly probable” in the absence of screening for Chorein blot or *VPS13A* mutations [7].

In conclusion, our study of 15 patients collected worldwide, is in favour of long-term efficacy and relative safety of the bilateral GPi-DBS for severe, drug-resistant hyperkinetic movement disorders (chorea, dystonia and/or orofacial movements and self-mutilations) in ChAc. DBS provides long-term motor and functional improvement but has less effect on long-term dependency. Also, as reported previously in NBIA patients [15] preoperative severity is predictive of a good response to bilateral GPi-DBS. Our study emphasizes the need to improve prospective data collection in international frameworks; gathering clinical characteristics of ChAc patients and their long term monitoring is essential to further evaluate interventions such as DBS in this debilitating disease.

## Supporting Information

Appendix S1 **Participating Centres.**
(DOCX)

Table S1 **Time-schedule of the data collected during the study.**
(DOCX)

Table S2 **Surgical procedures.**
(DOCX)

Table S3 **Electrode coordinates.**
(DOCX)

Table S4 **Optimal stimulation settings.**
(DOCX)
