# Supplementary material for: Short and Long Term Outcome of Bilateral Pallidal Stimulation in Chorea-Acanthocytosis
Source: PLoS One. 2013 Nov 5;8(11):e79241. doi: 10.1371/journal.pone.0079241 (PMC3818425; doi:10.1371/journal.pone.0079241)
Supplement: Appendix S1 — Participating Centres. (DOCX) [file pone.0079241.s001.docx]

**Appendix S1.** Participating Centres

Department of Neurology and department of Neurosurgery, Fundacion Jimenez Dıaz, Universidad Autonoma and CIBERNED, Madrid, Spain;

Department of Neurology, Hospital Donostia, San Sebastian, Spain;

Department of Neurology, Mount Sinai School of Medicine, New York, NY, USA;

Department of Neurology, Tokyo Metropolitan Neurological Hospital, Tokyo, Japan;

Movement Disorders Center, Division of Neurology, and Division of Neurosurgery, Toronto Western Hospital, University of Toronto, UHN, Toronto, Ontario, Canada;

Departments of Neurology, Samsung Medical Center, Sungkyunkwan University School of Medicine, Seoul, Korea;

Department of Clinical Neurophysiology, and Department of Neurosurgery, Centre Hospitalier Pellegrin, Bordeaux, France;

Department of Neurology, Alfred Hospital, Melbourne, Victoria, Australia;

Department of Neurology, Christian-Albrechts University, Kiel, Germany;

UCL Institute of Neurology, Queen Square, London, United Kingdom;

Department of Neurosurgery, West China Hospital of Sichuan University, Chengdu, Sichuan Province, China
