# Supplementary material for: Short and Long Term Outcome of Bilateral Pallidal Stimulation in Chorea-Acanthocytosis
Source: PLoS One. 2013 Nov 5;8(11):e79241. doi: 10.1371/journal.pone.0079241 (PMC3818425; doi:10.1371/journal.pone.0079241)
Supplement: Table S1 — Time-schedule of the data collected during the study. (DOCX) [file pone.0079241.s002.docx]

**Table S1.** Time-schedule of the data collected during the study

| **Study protocol** |
| --- |
| ***Preoperative assessment***  - Patient demographics  - Disease-specific findings:  Brain MRI status  Genetic testing (VPS13A gene analysis or Western blot for chorein)  Biological testing (laboratory values for acanthocytes and CK)  - Neurological examination, including:  Movement disorders severity (UHDRS-MS)  Functional status (UHDRS-IS, UHDRS-FCS)  Cognitive status (MMSE and/or other scales)  - Drug treatments  ***Intraoperative assessment***  - Surgery indication  - Surgical procedure  Electrode implantation  Target planning method  Anaesthesia  Use of frame (if so, frame type)  Trepanation procedure  Method of target localization  Microelectrode recording  Method of determination of lead location  Lead and implanted pulse generator (IPG) model used  - Optimal stimulator setting  - Adverse events  ***Early postoperative assessment and last outcome reporting***  - Neurological examination, including:  Movement disorders severity (UHDRS-MS)  Functional status (UHDRS-IS, UHDRS-FCS)  Cognitive status (MMSE and/or other scales)  - Optimal stimulator setting  - Drug treatments  - Adverse events |

UHDRS-MS = Unified Huntington’s Disease Rating Scale-Motor Score; UHDRS-IS = UHDRS-Independence Score; UHDRS-FCS = UHDRS Functional-Capacity Score; MMSE = Mini Mental State Examination
