# Supplementary material for: Short and Long Term Outcome of Bilateral Pallidal Stimulation in Chorea-Acanthocytosis
Source: PLoS One. 2013 Nov 5;8(11):e79241. doi: 10.1371/journal.pone.0079241 (PMC3818425; doi:10.1371/journal.pone.0079241)
Supplement: Table S2 — Surgical procedures. (DOCX) [file pone.0079241.s003.docx]

**Table S2.** Surgical procedures

| **P** | **Planning method** | **Anaesthesia** | **Frame** | **Type of opening** | **Target localization** | **Electrode recording, M-Stim** | **Location verification** | **Lead and IPG models** |
| --- | --- | --- | --- | --- | --- | --- | --- | --- |
| **1** | T, software, imaging | GA, throughout | CRW | BH | Electrophy, PP, software. | MER, 4 tracks each side | Post-op Im, VEP | 3387 Medtronic^®^ IPG: ND |
| **2** | PP, MRI, S&W | GA, throughout | CRW | BH | Electrophy, software | MER 3 tracks each side, M-Stim | Post-op MRI | 3387 Medtronic^®^, Activa PC^®^ |
| **3** | S&W, imaging | GA, throughout | RM | TDH | PP | No | Fluoroscopy, post-op Im | 3387 Medtronic^®^, Kinetra^®^ |
| **4** | PP, Software, imaging | LA with sedation throughout | Leksell | BH | Software, Electrophy | MER, M-Stim | Post-op Im, fluoroscopy | 3387 Medtronic^®^, Soletra^®^ |
| **5** | S&W, imaging | GA, throughout | Leksell | BH | Electrophy | MER, 2 tracks each side | Post-op Im | 3387 Medtronic^®^, Itrel II^®^ |
| **6** | S&W, imaging | GA, throughout | Leksell | BH | Electrophy | MER, 2 tracks each side | Post-op Im | 3387 Medtronic^®^, Itrel II^®^ |
| **7** | S&W, imaging | LA, neuroleptic anaesthesia | Leksell | BH | PP, software, Electrophy | MER, 1 track each side and M-Stim | Post-op Im, fluoroscopy | 3387 Medtronic^®^, Kinetra^®^ |
| **8** | 3D MRI, planning software | LA | Leksell | NA | PP, software, Electrophy | MER, M-Stim | Post-op Im | 3387 Medtronic^®^, Soletra^®^ |
| **9** | S&W, 3D MRI, software, PP, V | GA, intermittent | Leksell | BH | PP, Electrophy | MER, 5 tracks each side | Post-op Im | 3387 Medtronic^®^, Kinetra^®^ |
| **10** | PP, MRI-CT imaging fusion software | LA for electrodes, GA for lead and IPG | CRW | BH | PP, Electrophy | MER, 1 right pass, 2 left passes, M-Stim | Post-op Im | 3389, Medtronic^®^, Kinetra PC^®^ |
| **11** | S&W, PP, imaging, V | LA | Leibinger | BH | PP, Electrophy | M-Stim | Post-op Im | 3387 Medtronic^®^, Itrel II^®^ |
| **12** | Software, MRI | GA | Leksell | BH | Software | DIM | Stereotactic MRI | 3389 Medtronic^®^, Activa PC^®^ |
| **13** | Software, MRI | GA | Leksell | BH | Software | DIM | Stereotactic MRI | 3389 Medtronic^®^, Activa PC^®^ |
| **14** | Software, PP, imaging | LA throughout, GA for lead and stimulator | Leksell | BH | PP, Electrophy | M-Stim | Post-op Im, comparison to target | 3387 Metronic^®^, Kinetra^®^ |
| **15** | Software, PP, imaging | LA throughout, GA for lead and stimulator | Leksell | BH | PP, Electrophy | M-Stim | Post-op Im | 3387 Medtronic^®^, Kinetra^®^ |

BH = Burr Hole; CRW = Cosman-Roberts-Wells frame; DIM = Dynamic Impedance Monitoring; Electrophy = Electrophysiology; GA = General Anaesthesia; IPG = implanted pulse generator; LA = Local Anaesthesia; MER = microelectrode recording; M-Stim = Macro-Stimulation; NA = not available; P = patient; PP = Planning Platform; Post-op Im = post-operative Imaging; RM = Riechert-Mundinger frame; S&W = Schaltenbrand and Wahren atlas ; T = Talairach atlas ; TDH = Twist Drill Hole; V = ventriculography; VEP = visual-evoked potentials
