# Supplementary material for: Short and Long Term Outcome of Bilateral Pallidal Stimulation in Chorea-Acanthocytosis
Source: PLoS One. 2013 Nov 5;8(11):e79241. doi: 10.1371/journal.pone.0079241 (PMC3818425; doi:10.1371/journal.pone.0079241)
Supplement: Table S3 — Electrode coordinates. (DOCX) [file pone.0079241.s004.docx]

**Table S3.** Electrode coordinates according to the Schaltenbrand & Wahren atlas (1977) based on final intra-operative targeting

| **Patient** | **Electrode (contact 0) coordinates (mm)** | | |
| --- | --- | --- | --- |
|  | **X** | **Y** | **Z** |
| **1** | 21 | 3 | -4 |
| **2** | R = 20 | R = 2 | R = -4 |
|  | L = 21 | L = 3 | L = -5 |
| **3** | R = 19.9 | 2.9 | R = -2.9 |
|  | L = 19.5 |  | L = -4.1 |
| **4** | R = 21 | ND | ND |
|  | L = 20 |  |  |
| **5** | 17.5 | 2 | -5 |
| **6** | 23 | 2 | -5 |
| **7** | R = 19 | 1.4 | -3.9 |
|  | L = 19 |  |  |
| **8** | 21 | 3 | -3 |
| **9** | R = 15.3 | R = 3.0 | R = -5.6 |
|  | L = 15.4 | L = 5.2 | L = -5.3 |
| **10** | ND | ND | ND |
| **11** | R = 19.4 | 3.2 | -5 |
|  | L = -19.7 |  |  |
| **12** | ND | ND | ND |
| **13** | ND | ND | ND |
| **14** | R = 20 | R = 4 | R = 5 |
|  | L = 21 | L = 3 | L = 6 |
| **15** | R = 19 | R = 3 | R = 5 |
|  | L = 21 | L = 4 | L = 6 |
| **Mean** | 19.8 | 2.8 | -4.6 |

ND= Not Determined; R= Right; L=Left
