# Supplementary material for: Short and Long Term Outcome of Bilateral Pallidal Stimulation in Chorea-Acanthocytosis
Source: PLoS One. 2013 Nov 5;8(11):e79241. doi: 10.1371/journal.pone.0079241 (PMC3818425; doi:10.1371/journal.pone.0079241)
Supplement: Table S4 — Optimal stimulation settings. (DOCX) [file pone.0079241.s005.docx]

**Table S4.** Optimal stimulation settings

| **P** | **Pulse frequency** | | | **Pulse width** | | | **Pulse amplitude** | | | | **Contact settings in evolution** | | |
| --- | --- | --- | --- | --- | --- | --- | --- | --- | --- | --- | --- | --- | --- |
|  | intra-Op | EPOP | LOR | intra-Op | EPOP | LOR | intra-Op | EPOP | LOR | | intra-Op | EPOP | LOR |
| **1** | 140 | 130 | 180 | 120 | R 180 | 120 | 4 | 4.5 | 4.5 | | C+ R5- L2- | C+ R5- L2- | R5- L2- |
|  |  |  |  |  | L 130 |  |  |  |  | |  |  |  |
| **2** | 185 | 185 | 180 | 60 | 60 | R 120 | R 2.5 | R 3.0 | R 4 | | C+ R1- L5- | C+ R1- L5- | R9-11+ L1-3+ |
|  |  |  |  |  |  | L 90 | L 3.5 | L 3.8 | L 5 | |  |  |  |
| **3** | 130 | 145 | 130 | 120 | 120 | 60 | 2.6 | 2.3 | 2 | | C+1-5- | R4-5- L0-1- | R 4-5-6- L 0-1-2- |
| **4** | 50 | 40 | 40 | 210 | 120 | 150 | 3 | 2.5 | 2.8 | | C+1-2- | 1-2- | 1-3- |
| **5** | 130 | 130 | 160 | 60 | 60 | R 150 | 2 | 2 | R 3.5 | | C+1-2- | 0-1- | R0+1-2- L0-1-2+ |
|  |  |  |  |  |  | L 120 |  |  | L 3.3 | |  |  |  |
| **6** | 130 | 170 | 170 | 60 | 120 | 120 | 2.3 | R 2.6 | R 3.3 | | C+0-1- | 1-2- | 1-2- |
|  |  |  |  |  |  |  |  | L 2.6 | L 2.9 | |  |  |  |
| **7** | 40 | 130 | 185 | R 60 | 60 | 60 | R 3.5 | R 2.0 | R 2.9 | | M | R1- L4- | R2- L5- |
|  |  |  |  | L 120 |  |  | L 3.8 | L 3.8 | L 4.0 | |  |  |  |
| **8** | 130 | 130 | 130 | 90 | 90 | 90 | R 2.9 | R 2.9 | R 2.9 | | C+ 0- 4- | 0-, 4- | 0-, 4- |
|  |  |  |  |  |  |  | L 3.0 | L 3.0 | L 3.0 | |  |  |  |
| **9** | 40 | 40 | 130 | 90 | 90 | 90 | 3.5 | 3.5 | R 2.0 | | C+ R0-1- L4-5- | R4-5-L0-1- | R6- L2- |
|  |  |  |  |  |  |  |  |  | L 3.0 | |  |  |  |
| **10** | 100 | 140 | 140 | 60 | R 120 | R 12 | 4.5 | 2.6 | 2.6 | | C+1-5- | 1- 5- | 1- 5- |
|  |  |  |  |  | L 90 | L 90 |  |  |  | |  |  |  |
| **11** | 1 week micro-lesioning effect; STOP at 3 weeks with DBS, ineffective | | | | | | | | | | B | ND | NA |
| **12** | 130 | 130 | NA | 60 | 60 | NA | 2 | 2.5 | | NA | M | R1-, L9- | NA |
| **13** | 130 | 130 | NA | 60 | 90 | NA | 2 | 2.5 | | NA | M | R0- L9- | NA |
| **14** | 40 | 40 | 40 | 90 | 60 | 90 | 1.6 | 3.5 | | R 3.6 | C+0-4- | 0-, 4- | 0- 1-, 5- |
|  |  |  |  |  |  |  |  |  | | L 4.0 |  |  |  |
| **15** | 60 | 60 | 60 | 90 | 90 | 90 | 1.6 | 3.5 | | R 4.5 | C+0-4- | 1- 5- | 1-2-, 5-6- |
|  |  |  |  |  |  |  |  |  | | L 4.8 |  |  |  |

P = patient; intra-Op = intra-operative; EPOP = early post-operative; LOR = last-outcome report; R = right, L = left; M = Monopolar; B = Bipolar; NA = Not Available
